# Metabolic engineering of *Saccharomyces cerevisiae* for *de novo* biosynthesis of hydroxytyrosol and salidroside

**DOI:** 10.1128/aem.00712-25

**Published:** 2025-07-16

**Authors:** Jingfang Sun, Lingling Zhu, Liuping Duan, Furong Li, Shuai Guo, Jinlei Bian, Lin Yang

**Affiliations:** 1 School of Basic Medicine and Clinical Pharmacy, China Pharmaceutical University 56651, Nanjing, China; 2 School of Pharmacy, China Pharmaceutical University 56651, Nanjing, China; Kyoto University, Kyoto, Japan

**Keywords:** hydroxytyrosol, salidroside, tyrosol, phenylethanol compounds, *Saccharomyces cerevisiae*

## Abstract

Hydroxytyrosol and salidroside are phenylethanol compounds with significant industrial applications but limited availability due to low-yield natural extraction and complex chemical synthesis. In this study, *Saccharomyces cerevisiae* was engineered to achieve efficient *de novo* biosynthesis of these compounds. A tyrosol-producing strain (ZYT1) was optimized to produce 571.8 mg/L tyrosol, which served as the yeast chassis cell for hydroxytyrosol synthesis. By integrating *PaHpaB* and *EcHpaC*, strain ZYHT1 produced 304.4 mg/L hydroxytyrosol in shake-flask fermentation, which increased to 677.6 mg/L in a 15 L bioreactor after auxotrophic repair. For salidroside production, glycosyltransferase *RrU8GT33* was introduced into ZYT1, yielding strain ZYSAL1 with 48.4 mg/L salidroside. Enhancing UDP-glucose supply using truncated sucrose synthase (*tGuSUS1*) led to strain ZYSAL9+3, which achieved 1,021.0 mg/L in shake flasks and 18.9 g/L in fed-batch fermentation. This work demonstrates the scalable production of hydroxytyrosol and salidroside in yeast, providing a basis for industrial applications and advancing synthetic biology approaches for natural product biosynthesis.

**IMPORTANCE:**

Hydroxytyrosol and salidroside are valuable natural compounds with strong antioxidant, anti-inflammatory, and neuroprotective properties, widely used in pharmaceuticals, cosmetics, and health supplements. However, traditional extraction from plants is inefficient, and chemical synthesis is costly and environmentally unfriendly. In this study, we engineered *Saccharomyces cerevisiae*, a common yeast, to efficiently produce these compounds from simple carbon sources such as glucose and sucrose. By optimizing key biosynthetic pathways, improving cofactor supply, and enhancing sucrose metabolism, we achieved high production levels suitable for industrial applications. Our work provides a sustainable and scalable microbial platform for producing hydroxytyrosol and salidroside, reducing reliance on plant extraction and chemical synthesis. This research advances the field of microbial biotechnology by demonstrating how engineered yeast can serve as a green factory for valuable bioactive compounds, opening new possibilities for large-scale production and commercial use.

## INTRODUCTION

Polyhydroxyphenolic compounds, characterized by one or more hydroxyl groups attached to an aromatic ring, are widely used in fields such as pesticides, pharmaceutical intermediates, fragrances, dyes, synthetic resins, and antioxidants (1). These compounds generally exhibit excellent antioxidant properties, as the hydroxyl groups on the benzene ring can easily donate hydrogen electrons, thus exerting antioxidant effects. An increase in the number of hydroxyl groups typically enhances the reactivity of these compounds. Hydroxytyrosol and salidroside are representative phenylethanol compounds that have attracted significant attention due to their extensive biological activities and important applications.

Hydroxytyrosol (3,4-dihydroxyphenylethanol) is widely used in the food, cosmetic, and pharmaceutical industries (2, 3). However, the natural sources of hydroxytyrosol are primarily olives and grapes, which cannot meet the growing market demand. Current industrial production methods include extraction from olive leaves and chemical synthesis. These methods face challenges, such as complex reaction steps, low yields, and the generation of substantial wastewater, making them incompatible with the principles of green chemistry and sustainable development.

Biological synthesis, with its advantages of mild reaction conditions and lack of wastewater production, has become a promising mainstream method for hydroxytyrosol production. Existing biosynthetic strategies mainly fall into two categories: designing metabolic pathways to synthesize hydroxytyrosol from simple carbon sources (e.g., glucose or glycerol) and using structurally similar substrates (e.g., tyrosine or L-DOPA) to produce hydroxytyrosol via enzymatic catalysis. The former approach is often limited by the accumulation of byproducts and low yields, while the latter is hindered by high substrate costs or insufficient activity of key enzymes, posing challenges to industrial-scale production. Therefore, developing more cost-effective substrates and optimizing reaction pathways are crucial for the efficient biosynthesis of hydroxytyrosol.

Significant progress has been made in microbial hydroxytyrosol synthesis in recent years. For example, Li et al. engineered *Escherichia coli* to introduce hydroxylase HpaBC, constructing biosynthetic pathways using tyrosine and simple carbon sources as substrates, achieving hydroxytyrosol titers of 1,243 mg/L and 647 mg/L, respectively (4). Similarly, Liu et al. screened for the optimal combination of HpaB/HpaC enzymes and optimized their expression in *Saccharomyces cerevisiae*, resulting in a hydroxytyrosol titer of 6.9 g/L (5). The HpaBC system (HpaB hydroxylase and HpaC reductase) drives microbial aromatic compound degradation through redox coupling. HpaB oxygenase catalyzes aromatic ring hydroxylation, while HpaC functions as an NADH-dependent reductase supplying electrons to sustain HpaB’s activity. These advancements provide a solid foundation for further optimization toward the industrial production of hydroxytyrosol.

Salidroside is a water-soluble polyphenolic compound initially extracted and isolated from the roots and rhizomes of *Rhodiola* plants (6). Studies have shown that salidroside exhibits a variety of biological functions, including anti-tumor, anti-hypoxia, anti-inflammatory, and blood glucose-regulating properties. It holds great potential for the prevention and treatment of diseases, such as cerebral ischemia, cardiovascular conditions, and cancer (7–9). However, the natural production of salidroside is constrained by the growth conditions of *Rhodiola* plants, their low titers, and the difficulty of extraction, leading to a significant supply-demand imbalance. Additionally, chemical synthesis of salidroside is hindered by its structural complexity, lengthy synthesis steps, and high costs, which have so far prevented large-scale production (10).

In recent years, the rapid development of genetic and metabolic engineering technologies has opened new avenues for the microbial synthesis of salidroside. For instance, Xue et al. expressed a codon-optimized glycosyltransferase *UGT72B14* derived from *Rhodiola*, in *Escherichia coli*, achieving a salidroside titer of 6.7 mg/L (11). Bai et al. engineered *Escherichia coli* to construct a *de novo* pathway for salidroside synthesis, achieving a titer of 56.9 mg/L (12).

*Saccharomyces cerevisiae* has emerged as another important chassis organism for salidroside synthesis due to its metabolic flexibility and potential for pathway optimization. Jiang et al. constructed the first plasmid-free salidroside-producing strain by stepwise integration of key metabolic genes (*aro4*^*K229L*^*, aro7*^*G141S*^*, aroL, Pcaas,* and *Atugt85a1*) (13). The engineered strain incorporated the following: *aro4K229L*^*K229L*^ feedback-resistant mutant of *ARO4* encoding 3-deoxy-7-phosphoheptulonate synthase (*Saccharomyces cerevisiae*), *aro7G141S*^*G141S*^ feedback-resistant mutant of *ARO7* encoding chorismate mutase (*Saccharomyces cerevisiae*), aroL encoding shikimate kinase II (*Escherichia coli*), *PcAAS* tyrosine decarboxylase (*Petroselinum crispum*; GenBank: AAA33860.1), and A*tUGT85A1* encoding UDP-glycosyltransferase (*Arabidopsis thaliana*; GenBank: NM_102089). This multi-enzyme system achieved a salidroside titer of 732.5 mg/L. Guo et al. further optimized multiple pathways, increasing the titer to 1.82 g/L (14). Subsequently, Liu et al. enhanced *Saccharomyces cerevisiae* strains to achieve a salidroside titer of 26.55 g/L, the highest reported to date (15). However, large-scale production of salidroside has not yet been realized.

In *Rhodiola* plants, the biosynthesis of salidroside occurs in three stages: (i) the production of aromatic amino acids via the shikimate pathway, (ii) the conversion of aromatic amino acids to tyrosol, and (iii) glycosylation of tyrosol to form salidroside (16). Since microorganisms do not naturally produce salidroside, its synthesis relies on the design and construction of exogenous pathways. The study focuses on the metabolic engineering of *Saccharomyces cerevisiae* to systematically optimize the biosynthesis of tyrosol. Building on this foundation, hydroxylases or glycosyltransferases were introduced to enable the production of hydroxytyrosol or salidroside using glucose as the carbon source. The goal is to achieve efficient, green, and scalable industrial production, providing a novel solution to meet market demand.

## MATERIALS AND METHODS

### Reagents and standards

Tyrosol, salidroside, and hydroxytyrosol standards were purchased from Beijing Mreda Technology Co., Ltd. The DNA gel extraction kit and plasmid mini-prep kit were obtained from Tiangen Biological Technology. High-fidelity PCR enzyme PrimeSTAR GXL DNA Polymerase was purchased from TaKaRa Biotechnology (Dalian) Co., Ltd. Homologous recombinase 2 × ClonExpress, used for plasmid construction, was acquired from Vazyme Biotech Co., Ltd. The synthetic SD-URA medium, used for yeast genome integration screening, was sourced from Aili Biotechnology Co., Ltd. The primers used for plasmid construction are listed in Table S1. All codon-optimized genes (listed in Table S2) and primers were synthesized by GenScript Biotech Corp (Nanjing), and DNA sequencing was performed by Suzhou Genewiz, Inc.

### Strains, media, and culture conditions

The host strain used in this study was the yeast *Saccharomyces cerevisiae* CEN.PK2-1C (17). The growth medium was YPD (20 g/L peptone, 10 g/L yeast extract, and 20 g/L glucose). Liquid cultures were incubated at 30°C with shaking at 220 rpm, while colonies were grown at 30°C in a static incubator. For plasmid construction, the strain *Escherichia coli* TOP10F’ was used and cultured in LB medium (10 g/L peptone, 5 g/L yeast extract, and 10 g/L NaCl) at 37°C with shaking at 220 rpm or in a static incubator.

All gene knockouts, integrations, and other gene editing operations in this study were performed using the CRISPR-Cas9 system. Yeast transformations were performed using the lithium acetate method. Specifically, plasmids containing Cas9 protein and sgRNA, along with donor plasmids containing target genes and homologous arms, were co-transformed into yeast cells for genome integration and repair. Following transformation, cultures were plated on SD-URA plates and incubated statically at 30°C for three days. Single colonies were then picked for colony PCR verification.

### Plasmid and strain construction

All endogenous yeast genes, promoters, and terminators used in this study were amplified from the CEN.PK2-1C chassis strain. The exogenous glycosyltransferase gene *RrU8GT33* was derived from *Rhodiola rosea* and synthesized with codon optimization by GenScript Biotech Corp. The exogenous monooxygenase gene *PaHpaB* originated from *Pseudomonas aeruginosa*, and the reductase gene *EcHpaC* was from *Escherichia coli* BL21 (DE3). Additionally, the exogenous sucrose synthase gene *GuSUS1* and sucrose transporter gene *GlSUT4* were derived from *Glycyrrhiza* and *Solanum lycopersicum* cDNA, respectively. While CENPK2-1C exhibits high native invertase activity for extracellular sucrose hydrolysis (signal peptide-directed secretion), our engineered sucrose synthase operates intracellularly. This compartmentalization prevents functional overlap between the two systems. The gene IDs of the amplified genes are listed in Table S3.

The CRISPR plasmid pML104, used for gene editing, contained both a Cas9 expression cassette and a gRNA expression cassette. gRNA sequences were designed using the CHOPCHOP website (uib.no), and the sequences are provided in Table S4. gRNA fragments were prepared by annealing complementary oligonucleotide strands to form double-stranded DNA, which was then ligated into the pML104 plasmid to create CRISPR plasmids containing the desired gRNA.

The donor plasmid for gene knockout contained ~500 bp homologous arms flanking the target region and a linearized pUC19 backbone. For gene integration, the donor plasmid included ~500 bp homologous arms flanking the integration site, a yeast endogenous promoter, the target gene, a yeast endogenous terminator, and a linearized pUC19 backbone. All CRISPR and donor plasmids were first introduced into *Escherichia coli* TOP10F’ for cloning and sequencing verification, before being transformed into engineered *Saccharomyces cerevisiae* strains. The engineered strains and their genotypes constructed in this study are summarized in Table 1.

**TABLE 1:** Engineered strains constructed in this study

Strain	Genotype	Product
Cen.pk2-1c	*Mata; ura3-52, trp1-289, leu2-3, 112, his3Δ1; MAL2-8c; SUC2*	
ZYT2	Cen.pk2-1c, Δ*H2::P*_*TEF1*_*-TyrA*^*M53I/A354V*^*-T*_*GPD*_*, ΔH3::P*_*TEF1*_*-Bbxfpk-T*_*ADH1*_*, ΔP*_*TRP2*_*::P*_*YEN1*_*, Δpdc1, Δpha2*	Tyrosol
ZYT1	Cen.pk2-1c, *Δpdc1:: P*_*HXT7*_*-TKL1-T*_*ADH2*_*-P*_*TEF1*_*-RKI1-T*_*PGK1,*_*Δpha2::P*_*PGK1*_*-ARO2-T*_*GPD*_*-P*_*TEF1*_*-ARO10-T*_*PGK1*_*,Δ308a::P*_*TEF1*_*-ARO3-T*_*TDH2*_*,Δ416d::P*_*TDH3*_*-ARO4*^*K229L*^*-T*_*CYC1*_*-P*_*PGK1*_*-ARO7*^*G141S*^*-T*_*ADH1*_	Tyrosol
ZYHT1	ZYT1, Δ1622b:: *P*_*PGK1*_*-EcHpaC-T*_*ADH1*_*-P*_*TEF1*_*-PaHpaB-T*_*CYC1*_	Hydroxytrosol
ZYHT1 + 4	ZYHT1, ΔH7::*P*_*TRP1*_*-TRP1-T*_*TRP1*_*-P*_*LEU2*_*-LEU2-T*_*LEU2*_*-P*_*HIS3*_*-HIS3-T*_*HIS3,*_ *ΔH6::P*_*URA3*_*-URA3-T*_*URA3*_	Hydroxytrosol
ZYSAL1	ZYT1, ΔH1:: *P*_*TEF1*_*-RrU8GT33-T*_*PGK1*_	Salidroside
ZYSAL2	ZYT2, ΔH1:: *P*_*TEF1*_*-RrU8GT33-T*_*PGK1*_	Salidroside
ZYSAL3	ZYSAL1, ΔH2:: *P*_*TEF1*_*-TyrA*^*M53I/A354V*^*-T*_*GPD*_	Salidroside
ZYSAL4	ZYSAL1, ΔH3:: *P*_*TEF1*_*-Bbxfpk -T*_*ADH1*_	Salidroside
ZYSAL5	ZYSAL1, *ΔP*_*TRP2*_*::P*_*YEN1*_	Salidroside
ZYSAL6	ZYSAL1, *ΔTRP2*	Salidroside
ZYSAL7	ZYSAL1, ΔH4:: *P*_*TEF1*_*-ARO3*^*D154N*^*-T*_*TDH2*_	Salidroside
ZYSAL5 + 3	SAL5, ΔH7::*P*_*TRP1*_*-TRP1-T*_*TRP1*_*-P*_*LEU2*_*-LEU2-T*_*LEU2*_*-P*_*HIS3*_*-HIS3-T*_*HIS3*_	Salidroside
ZYSAL5 + 4	ZYSAL5 + 3, ΔH6:: *P*_*ura3*_*-ura3-T*_*ura3*_	Salidroside
ZYSAL8 + 3	SAL5 + 3, ΔH8:: *P*_*TEF1*_*-RrU8GT33-T*_*PGK1*_	Salidroside
ZYSAL9 + 3	ZYSAL8 + 3, ΔInt11:: *P*_*TEF1*_*-tGuSUS1-T*_*ADH1*_	Salidroside
ZYSAL9 + 4	ZYSAL9 + 3，ΔH6::*P*_*URA3*_*-URA3-T*_*URA3*_	Salidroside
ZYSAL10 + 3	ZYSAL9 + 3, ΔH5:: *P*_*TEF1*_ *-SlSUT4-T*_*ADH1*_	Salidroside
ZYSAL11 + 3	ZYSAL9 + 3, Δ1021b::*P*_*PGK1*_*-MAL11-T*_*TDH2*_	Salidroside
ZYSAL12 + 3	ZYSAL9 + 3, *ΔSUC2*	Salidroside
ZYSAL13 + 3	ZYSAL10 + 3, *ΔSUC2*	Salidroside
ZYSAL14 + 3	ZYSAL11 + 3, *ΔSUC2*	Salidroside

### Shake-flask fermentation

The yeast strain was streaked onto YPD solid medium and incubated statically at 30°C for 3 days. Five single colonies were picked from the plate and inoculated into 10 mL centrifuge tubes containing 4 mL of liquid YPD medium. The cultures were grown overnight with shaking at 220 rpm and 30°C. The following day, an appropriate volume of the overnight culture was inoculated into 100 mL Erlenmeyer flasks containing 20 mL of liquid YPD medium at an initial OD_600_ of 0.05. The cultures were incubated in shake flasks at 220 rpm and 30°C for 72 hours.

### Fed-batch fermentation in a 15 L bioreactor

The yeast strain was streaked onto YPD plates and incubated statically at 30°C for 3 days. Five single colonies were picked and inoculated into 10 mL centrifuge tubes containing 4 mL of liquid YPD medium, then cultured overnight at 220 rpm and 30°C to prepare the first-stage seed culture. The entire first-stage seed culture was transferred into 500 mL of liquid YPD medium and cultured at 220 rpm and 30°C for 24 hours to prepare the second-stage seed culture. The second-stage seed culture was then inoculated into an 8 L fermentation basal medium in a 15 L bioreactor at an initial inoculation volume of approximately 6%.

The fermentation basal medium consisted of YPD supplemented with 8 g/L KH_2_PO_4_, 3 g/L MgSO_4_, 0.72 g/L ZnSO_4_·7H_2_O, 10 mL/L trace element solution (including 5.75 g/L ZnSO_4_·7H_2_O, 0.32 g/L MnCl_2_·4H_2_O, 0.47 g/L CoCl_2_·6H_2_O, 0.48 g/L Na_2_MoO_4_·2H_2_O, 2.9 g/L CaCl_2_·H_2_O, and 2.8 g/L FeSO_4_· 7H_2_O, 80 mL 0.5 M EDTA, pH 6.0), and 12 mL/L vitamin solution (including 0.05 g/L biotin, 1 g/L pyridoxal HCl, 1 g/L calcium pantothenate, 25 g/L myo-inositol, 1 g/L nicotinic acid, 1 g/L thiamine HCl, and 0.02 g/L ρ-aminobenzoic acid). The initial stirring speed was set to 100 rpm, the aeration rate was maintained at 10 L/min, the tank pressure was held at 0.05 MPa, and the fermentation temperature was set to 30°C.

During fermentation, a feeding medium containing 500 g/L glucose, 9 g/L KH_2_PO_4_, 2.5 g/L MgSO_4_, 3.5 g/L K_2_SO_4_, 0.28 g/L Na_2_SO_4_, 10 mL/L trace element solution, 12 mL/L vitamin solution, 10 g/L yeast extract, and 20 g/L peptone was used for supplementation. Dissolved oxygen was maintained at 30%, and pH was kept constant at 5.5. Samples were taken every 12 hours to measure OD_600_ and perform liquid chromatography (LC) analysis.

### Liquid chromatography analysis

Take 1 mL of the fermentation broth and centrifuge at 12,000 rpm for 5 minutes. Filter the supernatant through a 2 µm membrane, and inject the filtrate into the LC system. Chromatographic analysis was performed by the Shimadzu LC-20A system with a WONDASIL C18 Chromatographic Column (5 µm, 4.6 mm × 250 mm).

The LC detection conditions for salidroside and tyrosol used an isocratic gradient of 15% methanol for 20 minutes, with a detection wavelength of 254 nm and a column temperature of 30°C. For hydroxytyrosol, the gradient was 25% methanol (0 minute), increased to 70% methanol (9 minutes), and returned to 25% methanol (11–15 minutes), with a detection wavelength of 280 nm and a column temperature of 30°C.

### Residual glucose analysis

Residual glucose was measured using a commercial glucose assay kit (Jiangxi Gelatins Biology Reagent Co., Ltd.) based on the glucose oxidase–peroxidase reaction. Every 4 hours, 200 µL of culture was sampled and centrifuged (12,000 × *g*, 5 minutes), and 20 µL of supernatant (diluted as needed) was mixed with 180 µL reaction reagent. After incubation at 25°C for 15 minutes, absorbance at 505 nm was recorded. A standard curve was constructed from known glucose standards, with ultrapure water as blank. All measurements were performed in technical triplicate.

### Statistical analysis

The data shown were expressed as the mean ± standard deviation (SD) from the representative of at least three sets of independent experiments. Significant differences between groups were determined by one-way or two-way ANOVA followed by post-hoc Tukey’s test by GraphPad Prism 9. * (*P* < 0.05), *** (*P* < 0.01), and *** (*P* < 0.001) indicate significant differences compared to the control group.

## RESULTS

### Construction of tyrosol-producing strains

**Fig 1:**
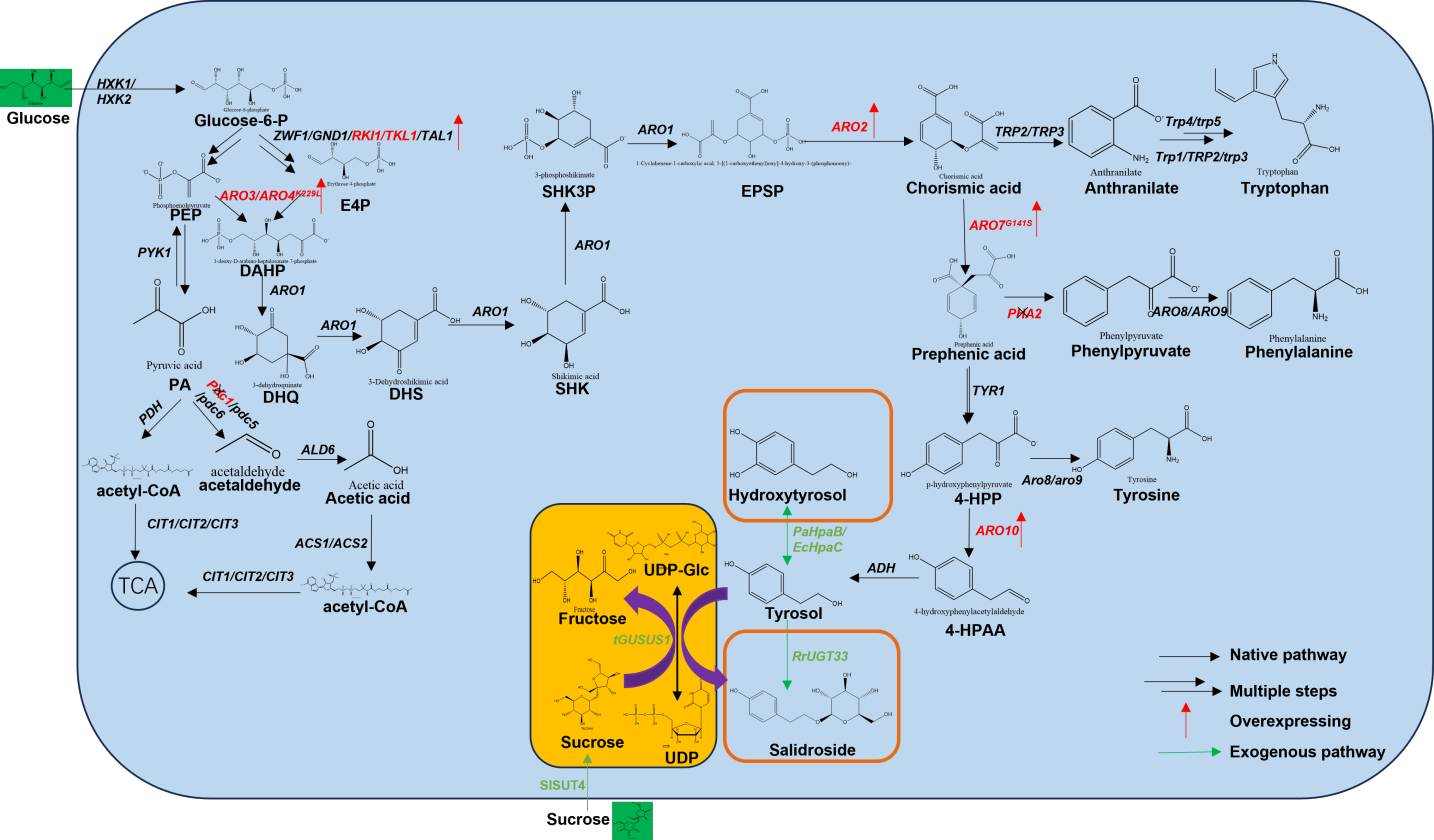
Synthesis pathways of tyrosol, hydroxytyrosol, and salidroside constructed in this study.

Tyrosol, an aromatic alcohol, is synthesized in *Saccharomyces cerevisiae* through the shikimate pathway followed by the Ehrlich pathway. To enhance the supply of erythrose-4-phosphate (E4P) and phosphoenolpyruvate (PEP), which are key precursors in the shikimate pathway, we overexpressed endogenous genes encoding ribose-5-phosphate isomerase (RKI1) and transketolase (TKL1) from the pentose phosphate pathway and introduced the phosphoketolase gene (Bbxfpk) from *Bifidobacterium breve* to increase PEP production. Phosphoketolase, a key enzyme in *Bifidobacterium*’s fructose-6-phosphate pathway, splits fructose-6-phosphate into acetyl phosphate and erythrose-4-phosphate. Its overexpression in yeast bypasses glycolysis (EMP), boosting carbon efficiency and metabolic flux (18). Additionally, the pyruvate decarboxylase gene (*PDC1*) was deleted to minimize the flux of pyruvate to acetaldehyde (Fig. 1). In *Saccharomyces cerevisiae*, deleting the main pyruvate decarboxylase gene PDC1 does not severely impact ethanol production because backup genes (*PDC5/6*) compensate to sustain the process (19).

ARO4 and ARO7 are feedback-controlled enzymes that restrict tyrosine synthesis. Mutations in ARO4^K229L^ and ARO7^G141S^ reduced this inhibition, resulting in a significant rise in extracellular aromatic amino acid levels in yeast (20, 21). To address the feedback inhibition of key enzymes in the shikimate pathway by tyrosine, we introduced feedback-resistant mutants of DAHP synthase (*ARO4*^*K229L*^) and chorismate mutase (*ARO7*^*G141S*^). Overexpression of *ARO3*, *ARO4*^*K229L*^, *and ARO7*^*G141S*^ significantly increased the production of extracellular aromatic amino acid metabolites.

To enhance the flux from prephenate to 4-HPP, the branched-chain amino acid transaminase gene (*ARO2*) was overexpressed, while the prephenate dehydratase gene (*PHA2*) was deleted to reduce the diversion of prephenate to phenylalanine. Furthermore, the tyrosol titer of ZYT1 was significantly higher than that of the wild-type strain, possibly due to the overexpression of ARO10 promoting the conversion of 4-HPP to 4-hydroxyphenylacetaldehyde (4-HPAA), a direct precursor of tyrosol.

**Fig 2:**
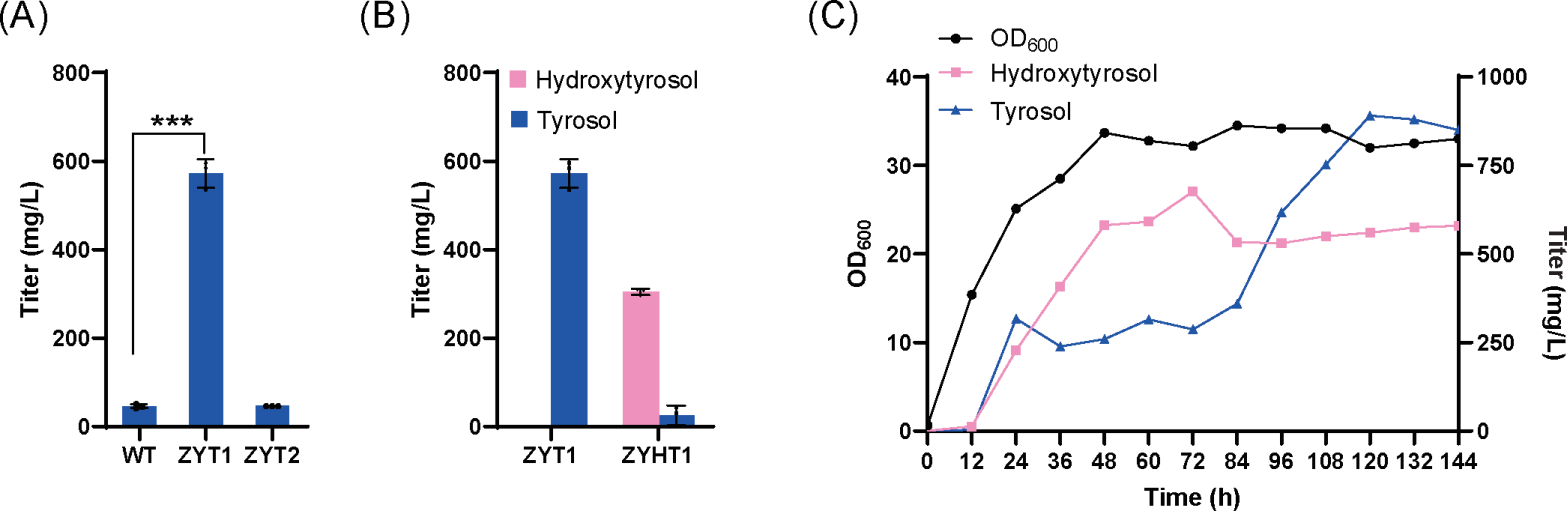
Production titers of tyrosol and hydroxytyrosol-producing strains. (A) Comparison of tyrosol production in different tyrosol-producing strains. (B) Hydroxytyrosol production in hydroxytyrosol-producing strains. (C) Fed-batch fermentation data of hydroxytyrosol-producing strains.

Further optimization of metabolic flux was achieved by deleting the TRP2 gene to reduce the diversion of chorismate to tryptophan and by deleting PHA2 to reduce the metabolic flux from prephenate to phenylalanine. Additionally, the introduction of a feedback-insensitive bifunctional enzyme (*EcTyrA*^*M53I/A354V*^) from *Escherichia coli* further increased 4-HPP synthesis. The final engineered strain, ZYT2, achieved a tyrosol titer of 46.9 mg/L after 72 hours of shake-flask fermentation. While the growth of ZYT2 was slightly lower than that of the wild-type strain, the production level was comparable to the parent strain CEN.PK2-1C, indicating successful rebalancing of metabolic flux (Fig. 2A).

**Fig 3:**
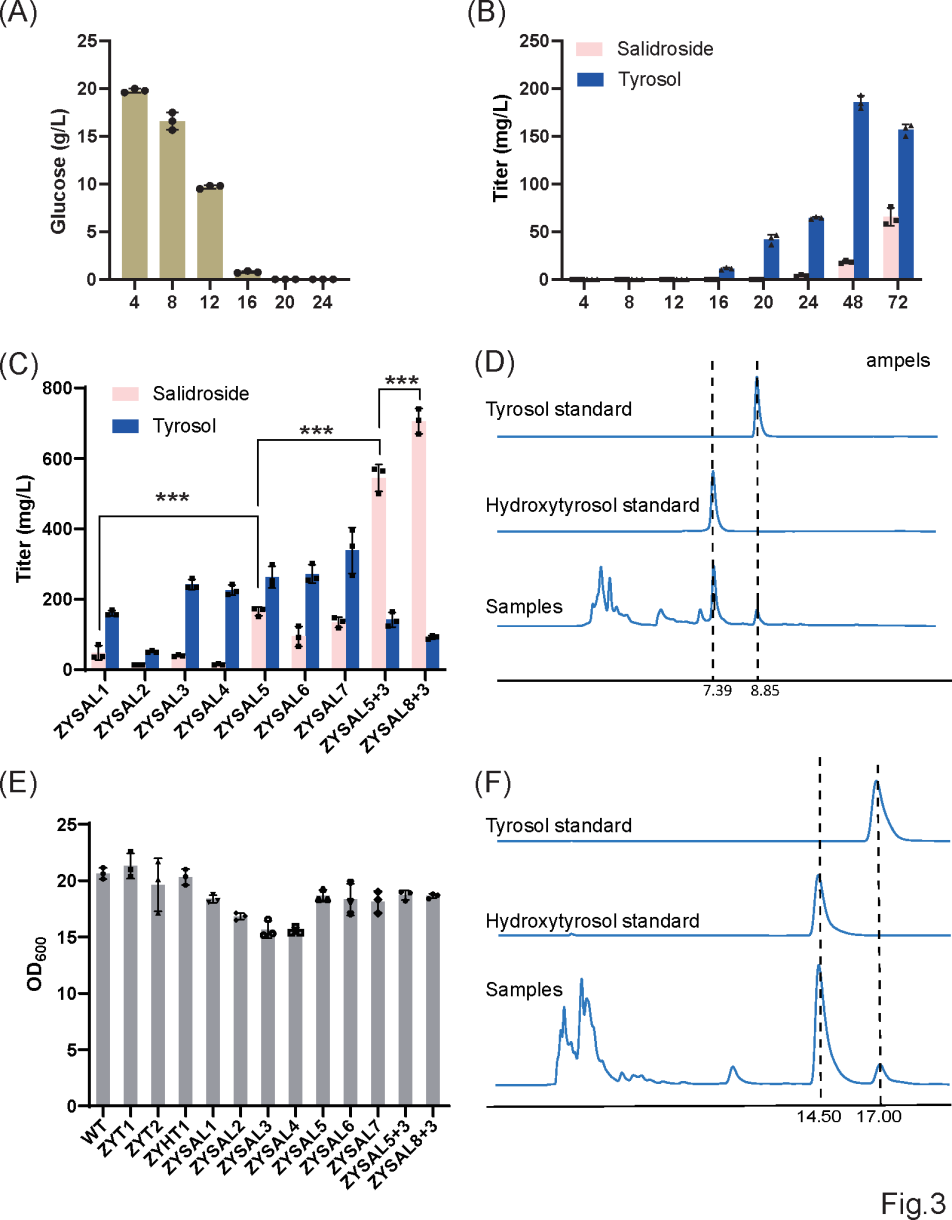
Production titers of salidroside-producing strains. (A) Glucose consumption profile of strain ZYSAL1 during shake-flask fermentation. (B) Time-course accumulation of salidroside by strain ZYSAL1 during shake-flask fermentation. (C) Comparison of salidroside and tyrosol production in salidroside-producing strains. (D) Representative LC chromatograms of tyrosol and hydroxytyrosol standards and samples using the hydroxytyrosol LC detection method. (E) OD_600_ of salidroside-producing strains after 72 hours of shake-flask fermentation. (F) Representative LC chromatograms of salidroside and tyrosol standards and samples using the salidroside LC detection method.

Following these modifications, the baseline tyrosol-producing strain ZYT1 was constructed using *Saccharomyces cerevisiae* CEN.PK2-1C as the chassis. Shake-flask fermentation for 72 hours demonstrated that ZYT1 produced 571.8 mg/L of tyrosol, which was approximately 12.5-fold higher than that of the wild-type strain (45.8 mg/L) (Fig. 2A). The OD_600_ of ZYT1 was slightly higher than that of the wild-type, with no significant difference observed (Fig. 3C). The comparison of results between ZYT2 and ZYT1 sufficiently demonstrates that overexpression of the shikimate pathway is critically necessary.

### Construction of hydroxytyrosol-producing strains

Hydroxytyrosol is a polyhydroxy aromatic compound synthesized by hydroxylating tyrosol at the 3-position. Previous studies demonstrated that co-expressing 4-hydroxyphenylacetate 3-monooxygenase (*HpaB*) and 4-hydroxyphenylacetate 3-reductase (*HpaC*) from different sources in *Saccharomyces cerevisiae* effectively produces hydroxytyrosol. Among various combinations, the *PaHpaB* gene from *Pseudomonas aeruginosa* and the *EcHpaC* gene from *Escherichia coli* showed optimal performance (5). Since tyrosol serves as the direct precursor for hydroxytyrosol, its production is critical for hydroxytyrosol accumulation. Using the high-tyrosol-producing strain ZYT1 as the chassis, the *PaHpaB* and *EcHpaC* genes were integrated into the genome to construct the hydroxytyrosol-producing strain ZYHT1. After 72 hours of shake-flask fermentation, the hydroxytyrosol titer in the supernatant reached 304.4 mg/L, with a concurrent accumulation of tyrosol at 25.0 mg/L (Fig. 2B).

To address the genetic deficiencies in leucine, tryptophan, histidine, and uracil biosynthesis in the *Saccharomyces cerevisiae* CEN.PK2-1C chassis, a complete gene expression cassette for these pathways was integrated into the ZYHT1 strain, resulting in the improved strain ZYHT1+4. Fed-batch fermentation using the ZYHT1+4 strain was performed in a 15 L bioreactor over six days. At the end of fermentation, the OD_600_ reached approximately 33. The final titers of hydroxytyrosol and tyrosol were 580 mg/L and 850 mg/L, respectively. Notably, hydroxytyrosol production peaked at 677.6 mg/L at 72 hours, accompanied by a tyrosol accumulation of 287.1 mg/L (Fig. 2C). These results highlight the enhanced production capacity of the engineered strain under optimized fermentation conditions.

### Construction of salidroside-producing strains

The synthesis of salidroside is catalyzed by a specific UDP-glucosyltransferase (UGT) through the glycosylation of the 8th hydroxyl group (-OH) on tyrosol using UDP-glucose as the glucose donor. Previous studies identified three UGTs capable of catalyzing the glycosylation of tyrosol at the 8th position from a pool of 34 UGTs derived from *Rhodiola* species. Among these, *RrU8GT33* demonstrated the highest efficiency for salidroside production (22). To construct a salidroside-producing strain, a codon-optimized *RrU8GT33* was integrated into the high-tyrosol-producing strain ZYT1, resulting in strain ZYSAL1. After 72 hours of shake-flask fermentation, ZYSAL1 produced 48.4 mg/L of salidroside, with a residual tyrosol accumulation of 160.5 mg/L. Similarly, RrU8GT33 was integrated into the ZYT2 strain, yielding ZYSAL2. ZYSAL2 produced 14.3 mg/L of salidroside and 51.6 mg/L of tyrosol after 72 hours, which were significantly lower than those of ZYSAL1 (Fig. 3A). Consequently, ZYSAL1 was selected for further optimization.

Shake-flask fermentations of strain ZYSAL1 were carried out for 72 hours, with residual glucose measured every 4 hours during the first 24 hours and every 24 hours thereafter. Glucose levels dropped from 19.76 g/L at 4 hours to 16.56 g/L at 8 hours, 9.68 g/L at 12 hours, 0.76 g/L at 16 hours, and became undetectable by 20 hours. Salidroside was first detected at 24 hours (3.56 mg/L), rose to 18.13 mg/L at 48 hours, and reached 65.40 mg/L at 72 hours (Fig. 3A and B). These data indicate that glucose was fully consumed around 20 hours, after which salidroside accumulation proceeded gradually, peaking at 72 hours. On this basis, a 72-hour fermentation period was adopted for all subsequent shake-flask experiments.

The bifunctional TyrA (chorismate mutase/prephenate dehydrogenase) mediates shared and tyrosine-specific biosynthesis steps. The *EcTyrA*^*M53I/A354V*^ mutants from *Escherichia coli* abolish tyrosine feedback control, enhancing tyrosol production (23, 24). To enhance carbon flux towards salidroside synthesis, *EcTyA*^*M53I/A354V*^ and BbXFPK were introduced into ZYSAL1, generating strains ZYSAL3 and ZYSAL4, respectively. After 72 hours of shake-flask fermentation, ZYSAL3 produced 40.0 mg/L of salidroside and 242.1 mg/L of tyrosol, while ZYSAL4 produced 15.1 mg/L of salidroside and 225.9 mg/L of tyrosol. Both strains exhibited slightly reduced salidroside titers compared to ZYSAL1, with increased tyrosol accumulation and minor growth impairments (Fig. 3C through F).

To improve the metabolic flux of branch acid towards prephenate, genes encoding phthalate synthase, which diverts carbon from branch acid to phthalic acid, were downregulated or knocked out in ZYSAL1, generating strains ZYSAL5 and ZYSAL6. After 72 hours, ZYSAL5 produced 165.3 mg/L of salidroside and 262.7 mg/L of tyrosol, while ZYSAL6 produced 94.4 mg/L of salidroside and 272.0 mg/L of tyrosol. ZYSAL5 showed the most significant improvement in salidroside production.

*S. cerevisiae ARO3* encodes a DAHP synthase for aromatic amino acid biosynthesis. Its activity is feedback-regulated by L-Phe, while the *Aro3*^*D154N*^ mutant retains catalytic function but evades this inhibition (25). Further enhancement was achieved by integrating *ARO3*^*D154N*^ into ZYSAL5 to construct ZYSAL7. After 72 hours of shake-flask fermentation, ZYSAL7 produced 134.7 mg/L of salidroside and 338.8 mg/L of tyrosol. Although salidroside production slightly decreased compared to ZYSAL5, the difference was not statistically significant, while tyrosol accumulation increased (Fig. 3A).

Since tyrosol accumulation consistently exceeded salidroside production, indicating suboptimal glycosylation efficiency, the *RrU8GT33* gene copy number was increased in ZYSAL5 after addressing auxotrophic deficiencies for tryptophan, leucine, and histidine, resulting in strain ZYSAL8+3. After 72 hours, ZYSAL8+3 produced 705.6 mg/L of salidroside and 92.0 mg/L of tyrosol, significantly outperforming ZYSAL5+3, which produced 544.5 mg/L of salidroside and 141.8 mg/L of tyrosol (Fig. 3A).

Using ZYSAL5+4, fed-batch fermentation was conducted in a 15 L bioreactor over seven days. At the end of fermentation, the OD_600_ reached 165. The final titers of salidroside and tyrosol were 7.6 g/L and 3.5 g/L, respectively. Peak salidroside production occurred at 156 hours, reaching 7.9 g/L, with a concurrent tyrosol titer of 3.6 g/L (Fig. S1).

### Enhancement of salidroside production by strengthening the UDP-glucose biosynthetic pathway

The synthesis of salidroside relies on a sufficient supply of UDP-glucose. This study sought to improve salidroside titers by introducing heterologous transporters and hydrolases to generate UDP-glucose from simple substrates such as sucrose. Although *Saccharomyces cerevisiae* can metabolize sucrose intracellularly and extracellularly, its native sucrose transport system exhibits low efficiency. Therefore, heterologous expression of sucrose transporters was employed to enhance sucrose utilization and fermentation performance.

Two genes were selected for heterologous expression: the sucrose transporter gene *SlSUT1* from tomato (*Solanum lycopersicum*) and the sucrose synthase gene *GuSUS2* from licorice (*Glycyrrhiza uralensis*). Sucrose synthase is a glycosyltransferase widely found in plants that catalyzes sucrose cleavage and synthesis. Based on the work of Zhang et al., truncation of the N-terminal flexible region of plant-derived sucrose synthase significantly improves its stability and activity (26). Thus, the truncated *tGuSUS1* was integrated into ZYSAL8+3 to create ZYSAL9+3. Considering the limited native sucrose transport capability of *Saccharomyces cerevisiae*, the tomato sucrose transporter gene *SlSUT4* was introduced into ZYSAL9+3 (27), resulting in the construction of strain ZYSAL10+3.

**Fig 4:**
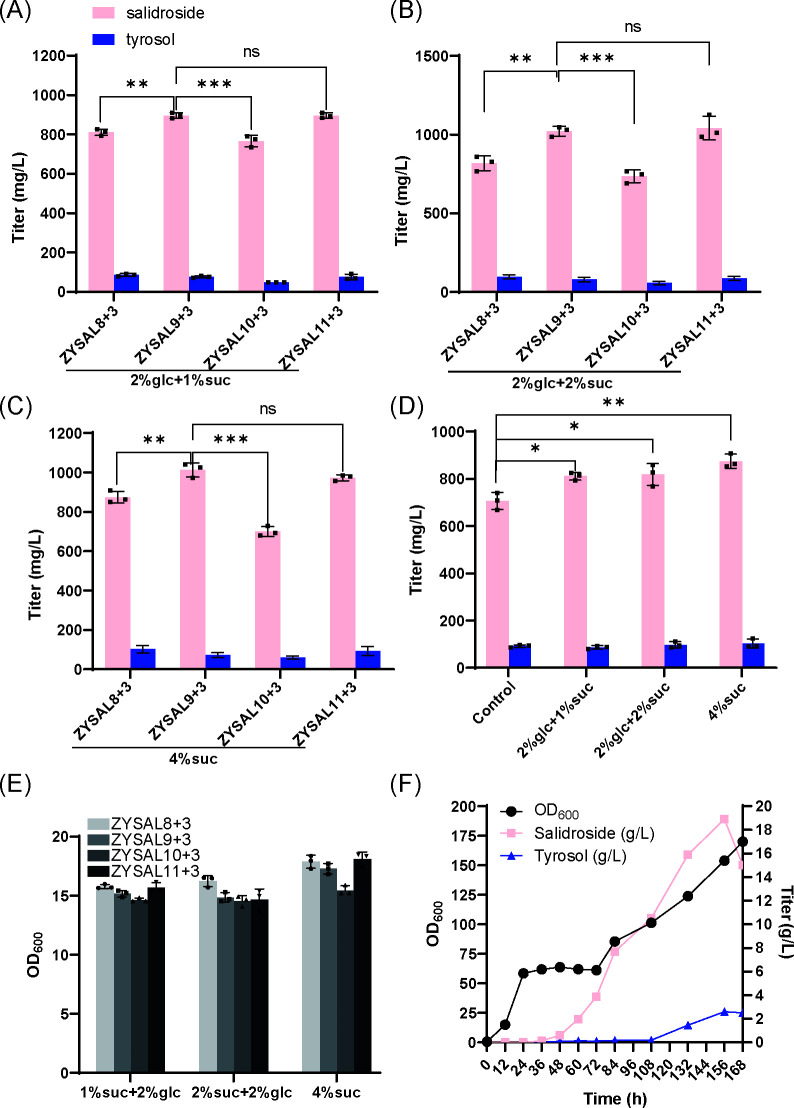
Effect of glycosyltransferase on salidroside accumulation. (A) Comparison of salidroside production in UDP-Glc-modified strains in 1% sucrose YPD medium during shake-flask fermentation. (B) Comparison of salidroside production in UDP-Glc-modified strains in 2% sucrose YPD medium during shake-flask fermentation. (C) Comparison of salidroside production in UDP-Glc-modified strains in 4% sucrose and glucose-free YPD medium during shake-flask fermentation. (D) Comparison of salidroside production in ZYSAL8+3 strains at different sucrose concentrations. (E) OD_600_ of UDP-Glc-modified strains during shake-flask fermentation at different sucrose concentrations for 72 hours. (F) Fed-batch fermentation data of salidroside-producing strains.

Shake-flask fermentation was performed to compare salidroside production among ZYSAL8+3 (control), ZYSAL9+3, and ZYSAL10+3 at sucrose concentrations of 1%, 2%, and 4%. At 1% sucrose, salidroside titers were 810.9, 895.7, and 766.4 mg/L for ZYSAL8+3, ZYSAL9+3, and ZYSAL10+3, respectively (Fig. 4A and E). At 2% sucrose, titers increased to 817.9, 1021.0, and 734.9 mg/L (Fig. 4B and E), while at 4% sucrose, titers were 802.5, 1013.0, and 699.5 mg/L (Fig. 4C and E), respectively. These results demonstrate that the introduction of *SlSUT4* did not enhance salidroside production but instead resulted in reduced titers compared to ZYSAL9+3, likely due to the inefficiency of *SlSUT4* in sucrose transport within yeast cells and potential inhibitory effects associated with heterologous gene expression. Sucrose is composed of glucose and fructose linked by an α-glycosidic bond. To further improve sucrose permeation, the endogenous α-glucoside transporter MAL11 was overexpressed in ZYSAL9+3, generating strain ZYSAL11+3. Shake-flask fermentation at 1%, 2%, and 4% sucrose concentrations showed salidroside titers of 895.5, 1041.0, and 973.3 mg/L, respectively, similar to those of ZYSAL9+3 under identical conditions.

Overall, ZYSAL9+3 exhibited the highest salidroside titers across sucrose concentrations. The truncated sucrose synthase tGuSUS1 significantly improved salidroside titers, while SlSUT4 suppressed salidroside accumulation, and MAL11 overexpression had no notable effect (Fig. 4A through C).

**Fig 5:**
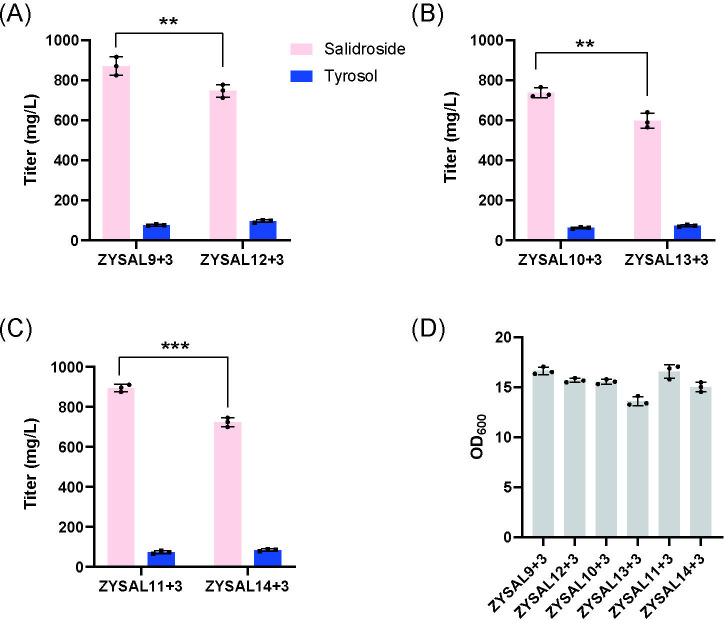
Effect of SUC2 deletion on salidroside titer in UDP-Glc–modified strains. All strains were fermented in shake flasks using YPD medium containing 1% sucrose and 2% glucose. (A) Salidroside titer in ZYSAL9+3 and ZYSAL12+3 (SUC2 deletion). (B) Salidroside titer in ZYSAL10+3 and ZYSAL13+3 (SUC2 deletion). (C) Salidroside titer in ZYSAL11+3 and ZYSAL14+3 (SUC2 deletion). (D) OD_600_ of SUC2-deleted strains after 72 hours of shake-flask fermentation.

Since the chassis strain *S. cerevisiae* CEN.PK2-1C expresses invertase *SUC2*, which hydrolyzes extracellular sucrose into glucose and fructose and limits intracellular sucrose uptake, we deleted *SUC2* in strains ZYSAL9+3, ZYSAL10+3, and ZYSAL11+3 to generate ZYSAL12+3, ZYSAL13+3, and ZYSAL14+3. These strains were cultivated in shake flasks containing YPD with 1% sucrose and 2% glucose for 72 hours, and salidroside titers were determined by HPLC. The SUC2 deletion strains produced 745.90 mg/L (ZYSAL12+3), 597.95 mg/L (ZYSAL13+3), and 723.15 mg/L (ZYSAL14+3) salidroside, all of which were lower than the corresponding parental strains under the same conditions (Fig. 5A through D). This suggests that invertase-mediated sucrose hydrolysis may still play a positive role in supporting carbon flux and energy supply under our current fermentation conditions, despite the spatial separation between invertase and glycosyltransferase. It is possible that the complete loss of periplasmic sucrose hydrolysis limits the availability of intracellular glucose, thereby reducing the metabolic precursors and overall production efficiency. Once again, reverse proof confirms that the sucrase pathway serves as the primary route for sucrose assimilation, and this pathway also plays a significant role in the synthesis of UDP-glucose.

To evaluate its industrial potential, ZYSAL9+3 was auxotrophically complemented to generate ZYSAL9+4 and was subjected to fed-batch fermentation in a 15 L bioreactor. After seven days, the OD_600_ reached 170, with final salidroside and tyrosol titers of 15 g/L and 2.5 g/L, respectively. Peak salidroside production was observed at 156 hours, reaching 18.9 g/L, while tyrosol concentration was 2.6 g/L (Fig. 4F). These results demonstrate that strengthening UDP-glucose supply through metabolic engineering significantly enhances salidroside production, providing a solid foundation for its industrial-scale production.

## DISCUSSION

This study established *Saccharomyces cerevisiae* as an efficient microbial platform for industrial-scale biosynthesis of hydroxytyrosol (677.6 mg/L) and salidroside (18.9 g/L), outperforming conventional methods in both shake-flask and bioreactor fermentations. The discussion below provides an in-depth analysis based on comparisons of various host systems, the limitations of yeast, and future metabolic optimization strategies.

### Comparison of production efficiency with other host systems

*Escherichia coli*, owing to its rapid growth and well-established genetic tools, has also been used for the synthesis of phenylethanol compounds. Previous studies have shown that by introducing the yeast-derived *ARO10* and *ADH6* and the Arabidopsis-derived glycosyltransferase *AtUGT85A1*, fed-batch fermentation in a 5 L bioreactor for 46 hours achieved a salidroside titer of 16.8 g/L, with a production rate of 0.4 g/(L·h) (28). In this study, glycosyltransferase *RrU8GT33* and truncated sucrose synthase *tGuSUS1* were integrated into *Saccharomyces cerevisiae*, and fed-batch fermentation in a 15 L bioreactor for 156 hours generated salidroside at a titer of 18.9 g/L. In comparison, the bacterial system exhibits superior intrinsic production efficiency relative to yeast. This difference may stem from the following factors: (i) Metabolic burden and growth rate: *Escherichia coli*, with its streamlined metabolic network, typically shows a faster growth rate and higher carbon flux toward aromatic compounds, whereas the eukaryotic complexity of yeast (such as compartmentalization and strict regulatory mechanisms) may restrict precursor supply. (2) Glycosylation efficiency: Glycosylation is a key step in the synthesis of salidroside, and *Escherichia coli* may have an advantage in utilizing exogenous glycosyltransferases and engineering UDP-glucose supply. Although this study employed RrU8GT33 and exogenous tGuSUS1 to enhance UDP-glucose supply and narrow the gap with the bacterial titer, limitations remain. In addition, the introduction of the tomato sucrose transporter *SlSUT4* resulted in a decreased titer, possibly due to insufficient sucrose transport efficiency of SlSUT4 and its inhibitory effect on UDP-Glc synthesis. Overexpression of the endogenous α-glucoside transporter *MAL11* also failed to significantly enhance the titer, indicating that MAL11 plays a limited role in sucrose transport in yeast. Other host systems, such as *Cyanobacteria* and *Bacillus subtilis*, generally exhibit titers lower than 200 mg/L due to their lack of glycosylation capabilities or limited UDP-glucose supply. In contrast, yeast shows a significant advantage in glycosylation modifications.

### Persisting limitations in yeast engineering‌

Although *Saccharomyces cerevisiae* has shown excellent performance in the synthesis of phenylethanol compounds, its limitations cannot be overlooked, as highlighted by the following issues:

- Uneven metabolic flux distribution: Yeast metabolism preferentially supports growth-related pathways. For example, in the early stage of salidroside synthesis, the strain ZYSAL1 achieved a titer of only 48.4 mg/L, mainly because UDP-glucose was competitively consumed for cell wall synthesis. By employing a truncated sucrose synthase (tGuSUS1) to specifically enhance UDP-glucose supply, the titer increased to 1,021.0 mg/L; however, this approach may interfere with other pathways, such as lipid synthesis, that also rely on the same precursor.
- Hydroxytyrosol toxicity and low tyrosol hydroxylation efficiency: We successfully constructed the strain ZYHT1 by using the hydroxylase PaHpaB from *Pseudomonas aeruginosa* and the reductase EcHpaC from *Escherichia coli*, enabling direct synthesis of hydroxytyrosol from glucose. The result of shake-flask fermentation showed that ZYHT1 produced hydroxytyrosol at a titer of 304.4 mg/L. However, after addressing nutrient limitations, fed-batch fermentation in a 15 L bioreactor only increased the titer to 677.6 mg/L, and the OD_600_ of ZYHT1 was merely 33. This may be attributed to the antimicrobial activity of hydroxytyrosol inhibiting cell growth. Additionally, the hydroxylation reaction catalyzed by PaHpaB/EcHpaC is highly dependent on NADPH, and the yeast pentose phosphate pathway (PPP) might be insufficient to meet this high energy demand, resulting in suboptimal hydroxylation efficiency. In the future, introducing the transhydrogenase *udhA* from *Bacillus subtilis* could help balance the NADPH/NADH ratio (29).
- Scale-up challenges‌: Although fed-batch fermentation increased the salidroside titer to 18.9 g/L, challenges, such as oxygen transfer and the accumulation of byproducts (e.g., ethanol), remain significant obstacles in large-scale yeast cultures. Enhancing oxygen tolerance and simplifying nutrient requirements could provide further opportunities to boost titers. Moreover, genetic stability during large-scale fermentation is a critical challenge. Although this study employed a genome integration strategy (e.g., strain ZYSAL9+3) to improve stability, its performance in cultures larger than 50 L still requires further validation.

### Further optimization strategies

To enhance the competitiveness of the yeast platform, future optimization efforts could focus on the following aspects:

- Dynamic pathway regulation (30, 31): Employ glucose-responsive promoters (e.g., HXT1) to activate the product synthesis pathway upon glucose depletion, effectively decoupling the growth and production phases while alleviating metabolic burden.
- Subcellular compartmentalization (32–34): Direct key enzymes to specific organelles, such as peroxisomes or mitochondria, to concentrate precursors and reduce competitive side reactions with cytosolic enzymes.
- Mixed microbial systems: Harness the complementary strengths of yeast and *Escherichia coli* by employing co-culture strategies, where *Escherichia coli* provides bacterial precursors and yeast facilitates glycosylation modifications. For example, in icariin synthesis, *Yarrowia lipolytica* executed the glycosylation step, while *Escherichia coli* supplied the precursors, leading to efficient production (35).
- Industrial applicability host: This study establishes yeast as a viable platform for the production of hydroxytyrosol and salidroside, and it is particularly well-suited for applications requiring eukaryotic post-translational modifications (such as functional glycosylation). However, for cost-sensitive large-scale production, *Escherichia coli* might still offer advantages. Additionally, exploring unconventional yeasts (such as *Yarrowia lipolytica*) to exploit their higher flux towards lipid-derived precursors for the synthesis of phenylethanol derivatives is also a promising endeavor.

## SUPPLEMENTAL MATERIAL

Supplemental material aem.00712-25-s0001.docx Tables S1 to S4; Figure S1.

ASM does not own the copyrights to Supplemental Material that may be linked to, or accessed through, an article. The authors have granted ASM a non-exclusive, world-wide license to publish the Supplemental Material files. Please contact the corresponding author directly for reuse.
