# Supplementary material for: Metabolic engineering of Saccharomyces cerevisiae for de novo biosynthesis of hydroxytyrosol and salidroside
Source: Appl Environ Microbiol. 2025 Jul 16;91(8):e00712-25. doi: 10.1128/aem.00712-25 (PMC12366309; doi:10.1128/aem.00712-25)
Supplement: Supplemental material — Tables S1 to S4; Figure S1. [file aem.00712-25-s0001.docx]

Table S1. The primer sequences used in this study.

| **Primers name** | **Sequence** |  | |
| --- | --- | --- | --- |
| pdc1 gRNA-F | TTTGGTCTCTGATCAGTCACCGTTACCCAAGGTGGTTTTAGAGCTAGAAATAGCAAGTT |  |  |
| pdc1 gRNA-R | TTTGGTCTCCTGCGCAAGCCCGGAATCGAA |  |  |
| pha2 308a gRNA-F | TTTGGTCTCGCGCAGGGGGATAGAGGCTGCTGGGGTTTTAGAGCTAGAAATAGCAAGTT |  |  |
| pha2 308a gRNA-R | TTTGGTCTCAAAACTATATTCTGTTTGACAAGTGTGCGCAAGCCCGGAATCGAAC |  |  |
| PUC19-F | TACCGAGCTCGAATTCACT |  |  |
| PUC19-R | AAGTCTGCAGGCATGCAAGCTTGGCGTAAT |  |  |
| pdc1 us-F | AAGCTTGCATGCCTGCAGACTTACATAGGAAAAAAAAATATATAAAC |  |  |
| pdc1 us-R | CCCGAAATTGTTCCTACGAGTGCAACAACAATTGCTTAGCTTG |  |  |
| HXT7p(TKL1)-F | CAAGCTAAGCAATTGTTGTTGCACTCGTAGGAACAATTTCGGGC |  |  |
| HXT7p(TKL1)-R | CTTATCAATGTCAGTGAATTGAGTCATTTTTTGATTAAAATTAAAAAAACTTTTTGT |  |  |
| TKL1-F | ACAAAAAGTTTTTTTAATTTTAATCAAAAAATGACTCAATTCACTGACATTGATAAG |  |  |
| TKL1-R | TATAAATCGTAAAGACATAAGAGATCCGCTTAGAAAGCTTTTTTCAAAGGAGAAATTAG |  |  |
| ADH2t(TKL1)-F | CTAATTTCTCCTTTGAAAAAAGCTTTCTAAGCGGATCTCTTATGTCTTTACGATTTATA |  |  |
| ADH2t(TKL1)-R | GGAGTAGAAACATTTTGAAGCTATGACTGTTTGTTGGAGGATGCCGTA |  |  |
| TEF1p(RIK1)-F | TACGGCATCCTCCAACAAACAGTCATAGCTTCAAAATGTTTCTACTCCT |  |  |
| TEF1p(RIK1)-R | CAATTTTTGGGACACCGGCAGCCATCTTAGATTAGATTGCTATGCTTTC |  |  |
| RKI1-F | GAAAGCATAGCAATCTAATCTAAGATGGCTGCCGGTGTCCCAAAAATTG |  |  |
| RKI1-R | GATCTATCGATTTCAATTCAATTCAATTCACTTTTCGGTAACTTCAACACTAC |  |  |
| PGK1t(RIK1)-F | GTAGTGTTGAAGTTACCGAAAAGTGAATTGAATTGAATTGAAATCGATAGATC |  |  |
| PGK1t(RIK1)-R | GCAGACATTCTGTGGAAAACAGTGACGCAGAATTTTCGAGTTATTAAAC |  |  |
| pdc1 ds-F | GTTTAATAACTCGAAAATTCTGCGTCACTGTTTTCCACAGAATGTCTGC |  |  |
| pdc1 ds-R | CGACGGCCAGTGAATTCGAGCTCGGTAGTGTTGAAATCAGACAACAAAGC |  |  |
| pha2 us-F | CCAAGCTTGCATGCCTGCAGACTTACATACTACCTTGCAGTTCCATTCT |  |  |
| pha2 us-R | GCAGATGTTATAATATCTGTGCGTAATTGAAGGATGAATGCGGAAAT |  |  |
| PGK1p(ARO2)-F | ATTTCCGCATTCATCCTTCAATTACGCACAGATATTATAACATCTGC |  |  |
| PGK1p(ARO2)-R | GCGGAACAGTTTCCCAAACGTTGACATTGTTTTATATTTGTTGTAAAAAGTAG |  |  |
| ARO2-F | TCTACTTTTTACAACAAATATAAAACAATGTCAACGTTTGGGAAACTGTTCCGC |  |  |
| ARO2-R | TAAATGCAAGATTTAAAGTAAATTCACTTAATGAACCACGGATCTGGAG |  |  |
| GPDt(ARO10)-F | CTCCAGATCCGTGGTTCATTAAGTGAATTTACTTTAAATCTTGCATTTAAAT |  |  |
| GPDt(ARO10)-R | AGGAGTAGAAACATTTTGAAGCTATGGAATCTGTGTATATTACTGCATCTAG |  |  |
| TEF1p(ARO10)-F | CTAGATGCAGTAATATACACAGATTCCATAGCTTCAAAATGTTTCTACTCCT |  |  |
| TEF1p(ARO10)-R | CTTTTCAATTGTAACAGGTGCCATCTTAGATTAGATTGCTATGCTTTCT |  |  |
| ARO10-F | AGAAAGCATAGCAATCTAATCTAAGATGGCACCTGTTACAATTGAAAAG |  |  |
| ARO10-R | CTATCGATTTCAATTCAATTCAATCTATTTTTTATTTCTTTTAAGTGCCGCTGC |  |  |
| PGK1t(ARO10)-F | GCAGCGGCACTTAAAAGAAATAAAAAATAGATTGAATTGAATTGAAATCGATAGA |  |  |
| PGK1t(ARO10)-R | TCTATACTAGTGTCGTTCTCCAATCGCAGAATTTTCGAGTTATTAAAC |  |  |
| pha2 ds-F | GTTTAATAACTCGAAAATTCTGCGATTGGAGAACGACACTAGTATAGATTATTCAG |  |  |
| pha2 ds-R | GTTGTAAAACGACGGCCAGTGAATTCGAGCTCGGTACCTAGCTTATCATTTATCGAATG |  |  |
| 308a us-F | ATTACGCCAAGCTTGCATGCCTGCAGACTTATATATGCAGAGAAGGAGCAAAT |  |  |
| 308a us-R | GGCAGTATTGATAATGATAAACTCGACGAAGTTAATGTTGAAATTTCACT |  |  |
| TDH3p(ARO4)-F | AGTGAAATTTCAACATTAACTTCGTCGAGTTTATCATTATCAATACTGCCAT |  |  |
| TDH3p(ARO4)-R | GCAGCGAACATTGGAGATTCACTCATTTTGTTTGTTTATGTGTGTTTATTCG |  |  |
| ARO4-F | CGAATAAACACACATAAACAAACAAAATGAGTGAATCTCCAATGTTCGCTGC |  |  |
| ARO4-R | ACTCCTTCCTTTTCGGTTAGAGCGGATCTATTTCTTGTTAACTTCTCTTC |  |  |
| CYC1t(ARO4)-F | GAAGAGAAGTTAACAAGAAATAGATCCGCTCTAACCGAAAAGGAAGGAGT |  |  |
| CYC1t(ARO4)-R | ATGTTATAATATCTGTGCGTCTTCGAGCGTCCCAAAACCTT |  |  |
| PGK1p(ARO7)-F | AAGGTTTTGGGACGCTCGAAGACGCACAGATATTATAACAT |  |  |
| PGK1p(ARO7)-R | CAGTTTCTGGTTTTGTGAAATCCATTGTTTTATATTTGTTGTAAAAAGTAG |  |  |
| ARO7-F | CTACTTTTTACAACAAATATAAAACAATGGATTTCACAAAACCAGAAACTG |  |  |
| ARO7-R | CATAAATCATAAGAAATTCGCTTACTCTTCCAACCTTCTTAGC |  |  |
| ADH1t(ARO7)-F | GCTAAGAAGGTTGGAAGAGTAAGCGAATTTCTTATGATTTATG |  |  |
| ADH1t(ARO7)-R | AGGAGTAGAAACATTTTGAAGCTATGCATATCTACAATTGGGTGAAAT |  |  |
| TEF1p(ARO3)-F | ATTTCACCCAATTGTAGATATGCATAGCTTCAAAATGTTTCTACTCCT |  |  |
| TEF1p(ARO3)-R | ACCGGCGTGATCGTTTTTAATGAACATCTTAGATTAGATTGCTATGCT |  |  |
| ARO3-F | AGCATAGCAATCTAATCTAAGATGTTCATTAAAAACGATCACGCCGGT |  |  |
| ARO3-R | CATTAAAGTAACTTAAGGAGTTAAATCTATTTTTTCAAGGCCTTTCTTC |  |  |
| TDH2t(ARO3)-F | GAAGAAAGGCCTTGAAAAAATAGATTTAACTCCTTAAGTTACTTTAATG |  |  |
| TDH2t(ARO3)-R | GTAATAGAAGTGGTAGCAATATGTAGCAAAGAGCGAAAAGCCAATTAGTGTGATACT |  |  |
| 308a ds-F | AGTATCACACTAATTGGCTTTTCGCTCTTTGCTACATATTGCTACCACTTCTATTAC |  |  |
| 308a ds-R | GTAAAACGACGGCCAGTGAATTCGAGCTCGGTACCCATCAACATATAGCTTAGATG |  |  |
| H1 us-F | ATTACGCCAAGCTTGCATGCCTGCAGACTTAGAGTAGTGTGCGTGAATGAAG |  |  |
| H1 us-R | GGAGTAGAAACATTTTGAAGCTATTGTTTCCGGGTGTACAATATGGACT |  |  |
| TEF1p(RrU8GT33)-F | GTCCATATTGTACACCCGGAAACAATAGCTTCAAAATGTTTCTACTCCT |  |  |
| TEF1p(RrU8GT33)-R | TGAGTGGTTTTTCAATTAAGCTCATTTTGTAATTAAAACTTAGATTAGATTGC |  |  |
| RrU8GT33-F | GCAATCTAATCTAAGTTTTAATTACAAAATGAGCTTAATTGAAAAACCACTCAC |  |  |
| RrU8GT33-R | GATCTATCGATTTCAATTCAATTCAATCTAACGGATATGTTTTGTTTTTGAG |  |  |
| PGK1t(RrU8GT33)-F | TCTCAAAAACAAAACATATCCGTTAGATTGAATTGAATTGAAATCGATAGAT |  |  |
| PGK1t(RrU8GT33)-R | GAACTTTACACTTCTCCTATGCACATATCGCAGAATTTTCGAGTTATTAAACT |  |  |
| H1 ds-F | AGTTTAATAACTCGAAAATTCTGCGATATGTGCATAGGAGAAGTGTAAAGTT |  |  |
| H1 ds-R | CCAGTGAATTCGAGCTCGGTAACAATCATGCTGACTCTGTGGC |  |  |
| YZ pdc1-F | ATTCGACAATGTTCTTGGTCT |  |  |
| YZ pdc1-R | ATGTCTTGTATCATCACCACCT |  |  |
| YZ pha2-F | AGTAGTAAAGGTGAGTAGGT |  |  |
| YZ pha2-R | CAGTTATCAGCATGACTCTTGTA |  |  |
| YZ 308a-F | GACACTGCTGAAAAAATTTCTG |  |  |
| YZ 308a-R | TATAGGTACTGTACTATAAGCG |  |  |
| YZ H1-F | TACTTGATAGCAAGACAGCAAACT |  |  |
| YZ H1-R | GTCTGTTTGAGTACGCTTTCAAT |  |  |
| TRP2 us-F | AAGCTTGCATGCCTGCAGACTTACCGGAGTGGCTCTCTTTATCAAT |  |  |
| TRP2 us-R | ATCTATCAATGCAACCGTTCAGTAACTGTGTACGTTAATGCCTTTAAC |  |  |
| YEN1p-F | GTTAAAGGCATTAACGTACACAGTTACTGAACGGTTGCATTGATAGAT |  |  |
| YEN1p-R | TGAATTTTGATGGAAGCGGTCATTTTCTTGTGCAGTATCCAGAAT |  |  |
| TRP2 ds-F | ATTCTGGATACTGCACAAGAAAATGACCGCTTCCATCAAAATTCAAC |  |  |
| TRP2 ds-R | ACGGCCAGTGAATTCGAGCTCGGTATGCCTCTGGAAGTCTTAGGACAT |  |  |
| YZ TRP2-F | CCTTTCAATCGTTGAAGTAGT |  |  |
| YZ TRP2-R | GTTGAATTTTGATGGAAGCGGT |  |  |
| H2 us-F | ATTACGCCAAGCTTGCATGCCTGCAGACTTTGCCATCACCATAAGCTGAT |  |  |
| H2 us-R | GGAGTAGAAACATTTTGAAGCTATTAAACTTCAACACCTTATATCATAAC |  |  |
| TEF1p(TyrA)-F | GTTATGATATAAGGTGTTGAAGTTTAATAGCTTCAAAATGTTTCTACTCCT |  |  |
| TEF1p(TyrA)-R | CGTAATGCGGTCAATTCAGCAACCATCTTAGATTAGATTGCTATGCTTTCT |  |  |
| TyrA-F | AGAAAGCATAGCAATCTAATCTAAGATGGTTGCTGAATTGACCGCATTACGCGA |  |  |
| TyrA-R | ATGCAAGATTTAAAGTAAATTCACTTACTGGCGATTGTCATTCGCCT |  |  |
| M53I-F | GAGCGCGAGGCATCTATCTTGGCCTCGCGTCGTGCAGAG |  |  |
| M53I-R | TCTGCACGACGCGAGGCCAAGATAGATGCCTCGCGCTCCG |  |  |
| A354V-F | AGCACTGGTTCGGCGATTACGTTCAGCGTTTTCAGAGTGAAAGCCG |  |  |
| A354V-R | CGGCTTTCACTCTGAAAACGCTGAACGTAATCGCCGAACCAGTGCT |  |  |
| GPDt(TyrA)-F | AGGCGAATGACAATCGCCAGTAAGTGAATTTACTTTAAATCTTGCAT |  |  |
| GPDt(TyrA)-R | ACTTCTTCTTCATGTAATAAACACAGGAATCTGTGTATATTACTGCATCT |  |  |
| H2 ds-F | GCAGTAATATACACAGATTCCTGTGTTTATTACATGAAGAAGAAGT |  |  |
| H2 ds-R | CCAGTGAATTCGAGCTCGGTATGAAAGAAGATCAAGCGAGTCCAT |  |  |
| YZ H2-F | GAGGACAGCTAGGTTTAT |  |  |
| YZ H2-R | CTGATGTGATGTGCAAGAT |  |  |
| H3 us-F | AAGCTTGCATGCCTGCAGACTTAGTTTCGTTGTTTCTTTTCATTAT |  |  |
| H3 us-R | AGGAGTAGAAACATTTTGAAGCTATAGAATATGCTGAATACTTGGTATATTAC |  |  |
| TEF1p(Bbxfpk)-F | GTAATATACCAAGTATTCAGCATATTCTATAGCTTCAAAATGTTTCTACTCCT |  |  |
| TEF1p(Bbxfpk)-R | GGGAGTTCCTATTACGGGATTTGTCATCTTAGATTAGATTGCTATGCT |  |  |
| Bbxfpk-F | AGCATAGCAATCTAATCTAAGATGACAAATCCCGTAATAGGAACT |  |  |
| Bbxfpk-R | CATAAATCATAAGAAATTCGCTTATTCGTTGTCCCCTGCGGT |  |  |
| ADH1t(Bbxfpk)-F | ACCGCAGGGGACAACGAATAAGCGAATTTCTTATGATTTATG |  |  |
| ADH1t(Bbxfpk)-R | ATTTTTCTTATAAATCATCCCTTCGCATATCTACAATTGGGTGAAAT |  |  |
| H3 ds-F | ATTTCACCCAATTGTAGATATGCGAAGGGATGATTTATAAGAAAAAT |  |  |
| H3 ds-R | GACGGCCAGTGAATTCGAGCTCGGTAGAACCAAGTTTACATATATATAT |  |  |
| YZ H3-F | AAAACGCGCGACTTCCTGTAAAT |  |  |
| YZ H3-R | GCGCTACAAGTGTGTCATAT |  |  |
| H7 us-F | ACACAGGAAACAGCTATGACCATGAACCTCCTTTTCGGCTTTTGAAGAT |  |  |
| H7 us-R | AATTGCGGGAAAGGACTGTGTTCCTTGTGATTGTTTATTTACATTTGG |  |  |
| His3-F | CAAATGTAAATAAACAATCACAAGGAACACAGTCCTTTCCCGC |  |  |
| His3-R | CTCTGAAATTAACAAAAAATTTCACTTGCCACCTATCACCACAAC |  |  |
| Leu2-F | GTTGTGGTGATAGGTGGCAAGTGAAATTTTTTGTTAATTTCAGAGGT |  |  |
| Leu2-R | GTTAACCGTTGATTTTTACTGATTCGTTGAGCCATTAGTATCAAT |  |  |
| Trp1-F | ATTGATACTAATGGCTCAACGAATCAGTAAAAATCAACGGTTAACGACAT |  |  |
| Trp1-R | TCTTTCTGCCGGAAAAATTAGCTAATTCCATTGCGGTGAAATGGT |  |  |
| H7 ds-F | ACCATTTCACCGCAATGGAATTAGCTAATTTTTCCGGCAGAAAGAT |  |  |
| H7 ds-R | CACGACGTTGTAAAACGACGGATCACTTGGAAGACATCACAAG |  |  |
| YZ H7-F | GAATGACGTTTGGTAAGGAAGT |  |  |
| YZ H7-R | AAGGGGACCAUGAUAUAACU |  |  |
| H6 us-F | ACAGGAAACAGCTATGACCATGTGACAATAAACTCTCTAATGGTG |  |  |
| H6 us-R | TATGGACCCTGAAACCACAGCCACTACTTCAGTACATCTCTCT |  |  |
| Ura3-F | AGAGAGATGTACTGAAGTAGTGGCTGTGGTTTCAGGGTCCAT |  |  |
| Ura3-R | ATATACACAGGGTCGAAACAAACAGCCTGTCTTATTGTTCTTGATTTG |  |  |
| H6 ds-F | CAAATCAAGAACAATAAGACAGGCTGTTTGTTTCGACCCTGTGTATAT |  |  |
| H6 ds-R | GTCACGACGTTGTAAAACGACGGGAACGGTTAGTTATGATCT |  |  |
| YZ H6-F | CAGAAACGACTTAGATCAC |  |  |
| YZ-H6-R | TCAGTTGTGCTAGTTCTAC |  |  |
| H6 gRNA-F | GATCAGGCGAAACACTTCATCCGG |  |  |
| H6 gRNA-R | AAACCCGGATGAAGTGTTTCGCCT |  |  |
| Int11 us-F | ATTACGCCAAGCTTGCATGCCTGCAGACTTGTGCTAGGCTATACTGTGCCAGAAT |  |  |
| Int11 us-R | AAGGAGTAGAAACATTTTGAAGCTATTTCAGTCGCTTTCGCTCATT |  |  |
| Int11 ds-F | TCCCCATTTCACCCAATTGTAGATATGCTCTTGTTCTTCTTGACGGAT |  |  |
| Int11 ds-R | ACGGCCAGTGAATTCGAGCTCGGTAGAATCGTCGATTTCCTTTTCTTC |  |  |
| tGuSUS1-F | CAATCTAATCTAAGTTTTAATTACAAAATGCACAGTCTCCGTGAGAGGCT |  |  |
| tGuSUS1-R | TAAAAATCATAAATCATAAGAAATTCGCTTACTCCTCAACAGCTAGGGGCACAG |  |  |
| TEF1p(tGuSUS1)-F | ATGAGCGAAAGCGACTGAAATAGCTTCAAAATGTTTCTACT |  |  |
| TEF1p(tGuSUS1)-R | GAGCCTCTCACGGAGACTGTGCATTTTGTAATTAAAACTTAGATTAGATTG |  |  |
| ADH1t(tGuSUS1)-F | CCTAGCTGTTGAGGAGTAAGCGAATTTCTTATGATTTATG |  |  |
| ADH1t(tGuSUS1)-R | GTATCATCCGTCAAGAAGAACAAGAGCATATCTACAATTGGGT |  |  |
| YZ Int11-F | CAATTGTGACAGTGATTGTGCT |  |  |
| YZ Int11-R | TATGTACAGGCAAAGAGAGT |  |  |
| H5 us-F | CAAGCTTGCATGCCTGCAGACTTCTGCAAACCCTTCTATACACTCACAT |  |  |
| H5 us-R | GAGTAGAAACATTTTGAAGCTATTGAAGCCAAAATAAAGGATTC |  |  |
| H5 ds-F | GTTTAATAACTCGAAAATTCTGCGCCTCATAAAGCACGTGGCCTCTTAT |  |  |
| H5 ds-R | ACGGCCAGTGAATTCGAGCTCGGTAAGACATTGTTTTATATTTG |  |  |
| SUT4-F | GCAATCTAATCTAAGTTTTAATTACAAAATGCCGGAGATAGAAAGGCAT |  |  |
| SUT4-R | TAAAAATCATAAATCATAAGAAATTCGCTCATGCAAAGATCTTGGGTCTCT |  |  |
| TEF1p-F | ATAGCTTCAAAATGTTTCTACTCCT |  |  |
| TEF1p-R | TTTGTAATTAAAACTTAGATTAGATTG |  |  |
| ADH1t-F | GCGAATTTCTTATGATTTAT |  |  |
| ADH1t-R | GCATATCTACAATTGGGTGAAAT |  |  |
| YZ H5-F | GACAAACGTCACAATTGATC |  |  |
| YZ H5-R | TCCAAATCTTGGACAGACAACT |  |  |
| SUC2 us-F | CCAAGCTTGCATGCCTGCAGACTTATGCTACGTTAGAAAGGCCCACAGT |  |  |
| SUC2 us-R | AGCCCTTTAGAATGGCTTTTGCATATACGTTAGTGAAAAGAAAAGC |  |  |
| SUC2 ds-F | CTTTTCTTTTCACTAACGTATATGCAAAAGCCATTCTAAAGGGCTTTAG |  |  |
| SUC2 ds-R | CGACGGCCAGTGAATTCGAGCTCGGTACATTCCCGATTGGAGTTCCTTCG |  |  |
| YZ SUC2-F | CGTCATTTAGAATAGTTTGTGAG |  |  |
| YZ SUC2-R | TAGCTTTATTTGTGCTCTCCT |  |  |

Table S2. The codon-optimized gene sequences synthesized in this study.

| **Gene** | **Sequence** | |
| --- | --- | --- |
| Bbxfpk | | ATGACAAATCCCGTAATAGGAACTCCCTGGCAAAAGTTAGACCGCCCGGTCTCTGAAGAAGCCATCGAGGGGATGGATAAATACTGGCGTGTTACCAACTATATGTCCATCGGTCAGATTTACCTGCGTAGCAACCCGCTGATGAAAGAGCCGTTTACCCGTGATGACGTCAAACATCGTCTTGTCGGTCATTGGGGCACGACTCCAGGTTTAAATTTTCTGTTGGCTCACATTAATCGTCTCATTGCGGACCATCAGCAAAACACCGTGTTTATCATGGGTCCGGGTCACGGCGGACCGGCAGGCACAAGCCAGAGCTACGTGGATGGCACCTACACCGAGTATTACCCGAACATTACCAAAGATGAGGCAGGCCTGCAAAAGTTCTTCCGCCAATTTTCCTATCCGGGTGGCATCCCTAGCCACTTTGCACCGGAGACGCCGGGTTCGATTCATGAAGGTGGTGAACTGGGCTATGCCTTATCGCACGCTTATGGTGCGGTCATGAATAACCCCAGCCTTTTTGTGCCGTGTATTATCGGTGACGGCGAGGCAGAAACCGGCCCACTTGCGACCGGTTGGCAGAGCAACAAGCTGGTGAACCCACGTACCGACGGCATCGTGCTGCCGATTCTGCACCTGAACGGCTATAAAATTGCCAACCCGACCATCTTGGCCCGCATCAGCGATGAAGAATTGCACGATTTTTTCCGTGGTATGGGTTACCACCCGTACGAGTTCGTGGCAGGCTTCGACAACGAGGACCACATGTCTATCCACCGCCGTTTTGCCGAGTTGTTCGAGACGATCTTCGATGAAATCTGCGACATCAAGGCGGCTGCGCAGACCGATGATATGACCCGTCCGTTTTATCCGATGTTGATTTTTCGTACCCCGAAAGGCTGGACGTGCCCGAAATTCATTGACGGTAAGAAGACCGAGGGCAGCTGGAGGGCGCACCAAGTTCCGCTGGCGTCTGCACGTGACACGGAAGAGCATTTCGAGGTGCTGAAGGGTTGGATGGAATCGTACAAGCCGGAAGAGCTGTTCAACGCCGACGGTTCTATCAAGGACGACGTGACGGCGTTCATGCCGAAGGGTGAACTGCGCATTGGTGCGAACCCGAACGCGAACGGCGGCGTGATTCGTGAGGATCTGAAGTTACCGGAATTGGACCAGTATGAAGTCACCGGCGTTAAAGAGTATGGTCACGGTTGGGGCCAAGTCGAAGCGCCAAGAGCGCTGGGTGCCTATTGCCGTGACATCATTAAGAATAACCCGGATTCCTTCCGAATCTTCGGTCCGGATGAGACGGCGAGCAATCGCCTGAATGCTACCTACGAGGTGACGGACAAACAATGGGATAACGGCTACCTCTCTGGTCTGGTAGACGAGCACATGGCAGTGACCGGTCAGGTGACAGAGCAGCTGTCTGAGCACCAATGCGAAGGTTTTCTGGAAGCGTACCTGCTGACCGGCCGTCATGGCATCTGGAGCAGCTACGAGAGCTTCGTTCATGTTATTGATAGCATGCTGAATCAGCATGCCAAATGGCTGGAAGCGACTGTGCGCGAAATTCCTTGGCGTAAACCGATTTCGAGCGTAAATCTGCTGGTTAGCTCCCACGTTTGGCGCCAGGACCACAATGGCTTTAGCCACCAAGATCCGGGCGTGACGAGCCTGTTGATCAACAAAACCTTTAACAACGACCACGTTACCAATATCTACTTCGCGACCGACGCGAACATGCTTCTGGCGATTAGCGAAAAATGTTTTAAAAGCACCAATAAGATCAATGCCATTTTTGCTGGTAAACAGCCGGCACCGACTTGGGTTACTTTGGACGAGGCGCGTGCTGAGCTGGAGGCTGGTGCGGCGGAATGGAAATGGGCCTCTAACGCTGAGAACAACGATGAGGTGCAAGTTGTCCTGGCGTCCGCTGGTGACGTTCCGACCCAAGAACTGATGGCGGCGTCGGATGCACTGAACAAGATGGGCATCAAATTCAAAGTTGTGAATGTGGTGGACTTGCTGAAGCTGCAATCCCGTGAAAACAATGATGAGGCACTGACCGATGAGGAGTTCACCGAATTGTTCACCGCTGACAAGCCGGTTCTGTTCGCGTATCATAGCTACGCTCAAGACGTTCGTGGTTTGATTTACGACCGTCCAAACCATGATAACTTTCACGTGGTTGGCTATAAAGAACAGGGTAGCACCACGACTCCGTTCGATATGGTTCGCGTGAATGATATGGACCGTTATGCGCTGCAAGCGGCGGCGCTCAAGTTAATCGACGCGGATAAGTACGCGGATAAGATCGACGAGTTGAATGCATTCCGCAAAAAGGCGTTTCAGTTTGCAGTTGATAACGGTTACGATATTCCGGAGTTCACCGACTGGGTTTATCCGGACGTTAAGGTGGACGAGACGCAGATGCTCTCAGCTACCGCAGCTACCGCAGGGGACAACGAATAA |
| RrU8GT33 | | ATGAGCTTAATTGAAAAACCACTCACGGCCATAGAGACTCGTGAAAAACCACACGCTGTGTGCATCCCATACCCAGCTCAAGGCCATATCAATCCCATGATGCAACTTGCAAAGCTCCTCCACCACTCTGGTTTCCACATAACGTTTGTCCACACTGAGTATAATTATGACCGTCTAGTGAAGTCTCAAGGTTCAGCTTGTGTGGCTGGTTTACCGGATTTCCGCTTTGAAGCCATCCCAGATGGCTTGCCCTCGACGAATGGTGATGTTACTCAAGACATTCCTCTGTTGAGTAGCTCTACTTCTAAAACCTGCTTGAAGCCGTTTAAGGAGTTATTGAAGAGGTTGCAGGACAAATGCAAAGAGTTACCTGATGATGTTCCGCCTCTGTCGTGCATCGTGTCTGATGCAGCCATGTCGTTTACGATCGATGCATCTGAGGAGTTTGGAGTGCCCATAGCGCTTCTTTGGACTGCAAGTGCCTGCGGGTTCTTGGGTTACACGCATTACCCATATCTAATTGACAGAGGTGTCATCCCATTGAAAGATGAGAGCCAATTAACAAACGGATACCTAGATATGAGCATAGATGGCATACCTTGTATGGAAGGTATCCGCTTACGAGACCTCCCAAGCTTTCTACGCACAACTGATTTAGATGATATGATGTTTAGTTATATACTGCACGAAATAAAACAAGTTTCAAGAGGCAGTGCTATCATTCTGAACACCTTTGAAGCTTTGGACCATGATGTCTTGGATAGTCTCTCCAAAATTTACCAAAATGTCATCCTGCCAGTTGGCCCTCTACATGTCTCGCTCAACAAGATCCCAAAACACTACCCACTTCAATCTTTAAGCTCGAATTTATGGAAAGATGACACAGACTGCATTCCCTGGCTGAGCTCTAAGGCTTCAAAATCAGTTATATACGTTAACTTTGGGAGCATCACGACGGTATCACCAAAACAAATTGTGGAGTTTGCGTGGGGATTGGCTAACAGCAAACACCCTTTCCTTTGGATAATCAGACCGGACTTGGTGGCAGGTGAGGCATCCATCATTCCGCAGGACTTCATGGATGAAACAAAAGGAAGAGGTTTGTTGGCTGGTTGGTGTGACCAAGAGCTTGTTCTCAACCATCCATCCATTGGAGGGTTTCTTACGCACTGTGGCTGGAACTCAATTATTGAAAGCATTAGCGCAGGAGTCCCTACGGTCTGCTGGCCATTTTTTGCTGAGCAGCAAACAAATTGTTGGTTTGCTTGCAAAAAATGGTGCATTGGGATGGAGATGCATACTGATGTAAAGAGGGATGAGGTTGACAAGCTGTTGAGAGAGCTAATGGAAGGTGACAAAGGGGAGGAGTTGAAGAGGAAGGCAACCAACTGGAAGAGGCTGGCAGAAGAAGCTGTTTCCTCCACTGGCTTATCAACCTTAAACTTCAGGACGTTAGTGAATCAAGTCCTGCTCTCAAAAACAAAACATATCCGTTAG |

Table S3. The gene IDs of the amplified genes.

| **Gene** | **GenBank ID** |
| --- | --- |
| *RKI1* | 854262 |
| *TKL1* | 856188 |
| *ARO3* | 851605 |
| *ARO4* | 852551 |
| *ARO7* | 856173 |
| *ARO2* | 852729 |
| *ARO10* | 851987 |
| *PaHpaB* | CP050330.1 |
| *EcHpaC* | CP026939.2 |
| *EcTyrA* | 947115 |
| *GuSUS1* | MK420465 |
| *GlSUT4* | NM_001247415 |

Table S4. The target sites and sgRNA sequences used for CRISPR experiments.

| Locus | Sequence |
| --- | --- |
| PDC1 | AGTCACCGTTACCCAAGGTG |
| PHA2 | GGGGGATAGAGGCTGCTGGG |
| 308a | CACTTGTCAAACAGAATATA |
| H1 | CCCCCCTTCTCTACTAGCAT |
| H2 | GAGATAGGTAAATAAACGCG |
| H3 | CGTGATTTATACAAAAGAAG |
| H5 | TGGCCCTGATAATAGTATGA |
| H8 | CTTGCTGGTACTCTGGGAAAA |
| TRP2 | TCAGTGCTAATTATCGATTG |
| 1622b | GTCACGTTCCTGAGGTTACT |
| Int11 | GAACAAGAACAACAAACTCC |


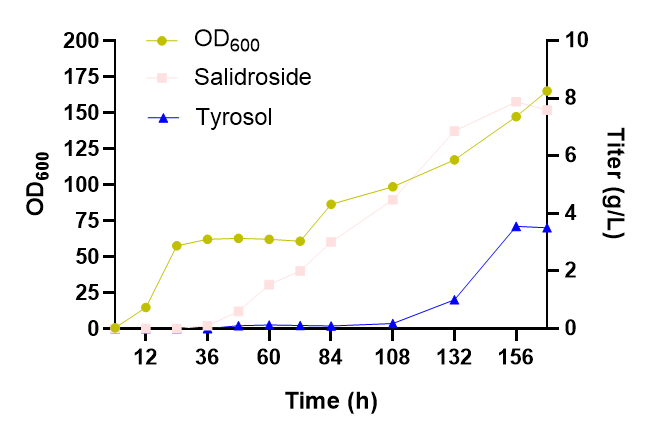


Figure S1: Fed-batch fermentation data for strain ZYSAL5+4.
